# Immunophenotypical Characterization of Limbal Mesenchymal Stromal Cell Subsets during In Vitro Expansion

**DOI:** 10.3390/ijms25168684

**Published:** 2024-08-09

**Authors:** Sara Aghazadeh, Qiuyue Peng, Fereshteh Dardmeh, Jesper Østergaard Hjortdal, Vladimir Zachar, Hiva Alipour

**Affiliations:** 1 Regenerative Medicine, Department of Health Science and Technology, Aalborg University, 9260 Gistrup, Denmark; saraag@hst.aau.dk (S.A.); qp@hst.aau.dk (Q.P.); feda@hst.aau.dk (F.D.); vlaz@hst.aau.dk (V.Z.); 2 Department of Ophthalmology, Aarhus University Hospital, 8200 Aarhus, Denmark; jesper.hjortdal@clin.au.dk

**Keywords:** limbal mesenchymal stem cells, sub-population, wound healing, immune regulation, corneal regeneration

## Abstract

Limbal mesenchymal stromal cells (LMSCs) reside in the limbal niche, supporting corneal integrity and facilitating regeneration. While mesenchymal stem/stromal cells (MSCs) are used in regenerative therapies, there is limited knowledge about LMSC subpopulations and their characteristics. This study characterized human LMSC subpopulations through the flow cytometric assessment of fifteen cell surface markers, including MSC, wound healing, immune regulation, ASC, endothelial, and differentiation markers. Primary LMSCs were established from remnant human corneal transplant specimens and passaged eight times to observe changes during subculture. The results showed the consistent expression of typical MSC markers and distinct subpopulations with the passage-dependent expression of wound healing, immune regulation, and differentiation markers. High CD166 and CD248 expressions indicated a crucial role in ocular surface repair. CD29 expression suggested an immunoregulatory role. Comparable pigment-epithelial-derived factor (PEDF) expression supported anti-inflammatory and anti-angiogenic roles. Sustained CD201 expression indicated maintained differentiation capability, while VEGFR2 expression suggested potential endothelial differentiation. LMSCs showed higher VEGF expression than fibroblasts and endothelial cells, suggesting a potential contribution to ocular surface regeneration through the modulation of angiogenesis and inflammation. These findings highlight the heterogeneity and multipotent potential of LMSC subpopulations during in vitro expansion, informing the development of standardized protocols for regenerative therapies and improving treatments for ocular surface disorders.

## 1. Introduction

The limbal epithelial stem cells (LESCs) reside in the limbal niche and are responsible for corneal epithelial integrity, transparency preservation, and regeneration [1,2,3]. The interactions among the limbal niche cells and extracellular matrix (ECM) components play a critical role in balancing the degeneration and regeneration mechanisms [4,5,6,7,8]. The stemness of LESCs’ is impacted by the limbal mesenchymal stem/stromal cells (LMSCs) involved in corneal regeneration [7,9,10], and the limbal melanocytes (LMs), which protect the LESCs from oxidative stress [11,12]. LMSCs are located underneath the basement membrane in the stroma [7,10] and are in close interaction with LESCs [7] throughout various signaling pathways including aquaporin-1 and vimentin [13], SDF/CXCR4 [14], BMP/Wnt [15], and IL-6/STAT3 [16], emphasizing their crucial role in ocular surface homeostasis. Additionally, LMSCs have been proposed as a suitable candidate for regenerative therapies for ocular surface treatment [17,18,19].

Corneal blindness ranks as the fourth most common cause of blindness worldwide (5.1%) [20,21]. Corneal defects can occur due to genetic predisposition, autoimmune disease, trauma, and recurrent infections following cataracts, glaucoma, and age-related macular degeneration as the main causes of visual impairment [20,21]. Current treatment strategies for corneal surface defects include conjunctival autografts [22], kerato-limbal allografts [23], LESCs or non-limbal epithelial cells like oral mucosa [24] or conjunctival epithelial cells [25] transplantations and supporting limbal niche restoration using biological factors [26,27,28]. While these treatments can improve corneal regeneration, there is a risk of graft rejection [29], and some cases require multiple treatment sessions to reach the desired results, increasing treatment costs significantly [30,31].

Mesenchymal stem/stromal cells (MSCs) have been used in regenerative therapies for several different health problems, including ocular surface treatment [19] due to their multipotency, low immunogenicity, immunomodulatory [32,33], and paracrine actions [34]. Several animal studies and clinical trials have also assessed the potential of bone marrow (BM) MSCs and adipose-derived MSC (ASC) in chemical burn [17,18,35], dry eye syndrome [36,37,38] and limbal stem cell deficiency (LSCD) [32,39,40] with different outcomes.

The International Society of Cellular Therapy (ISCT) has generally characterized MSCs by a fibroblast-like morphology, the capacity to adhere to plastic surfaces, and the expression of a trio of a cluster of differentiation (CD) molecules (CD73, CD90, and CD105) [41,42], while the literature highlights some discrepancies regarding their biological properties [43]. An in vitro study by Peng et al. (2020) demonstrated that all adipose-derived MSCs (ASCs) also expressed canonical markers CD29, CD166, and CD201 [44]. The additional characterization of ASCs based on their surface marker expressions during earlier and later in vitro culture stages (passages one to eight) demonstrated predominant lineages based on the expression of CD34, CD200, and CD271 after eight passages [44]. Different gene expression profiles can result in different phenotypical and functional properties [41,45,46,47,48]. Thus, such distinct subpopulations could indicate different biological functions and explain the reported functional variation in MSCs within a tissue-derived primary culture [49,50,51]. Furthermore, despite reported similarities in vitro, MSCs from different sources may not present comparable biological properties in vivo [52].

LMSCs express the typical characteristics of MSCs [10,53,54,55]. However, similar cell types from different organs may also not be comparable, presenting different biological properties or potential niche-specific limitations [52]. For example, BM-MSCs were shown to release paracrine growth factors and cytokines, including the stem cell factor (SCF), stromal cell-derived factor (SDF-1 or CXCL12), bone-morphogenetic protein 4 (BMP-4), and transforming growth factor beta (TGF-β) [56]. In contrast, LMSCs exhibit a more pronounced secretion of transforming growth factor alpha (TGF-a), epiregulin (EREG), amphiregulin (AREG), hepatocyte binding epidermal growth factor (HB-EGF), growth factor receptor bound protein 14 (GRB14), fibroblast growth factor 11 (FGF11) and cytokines including (C-X-C motif) ligand 1 (CXCL1), and CXCL2 [57,58].

Cell-based regenerative therapies can potentially revolutionize the treatment of diseases. However, moving the state of the art in cell therapies from the bench to the bedside requires a clear understanding and definition of the underlying processes. On this path, the complete characterization and profiling of cells based on their surface markers and gene expressions can identify superior cell sub-populations as a step towards developing standardized clinical protocols for different applications in regenerative therapies.

While MSCs have received considerable research focus, leading to the identification of several sub-populations with different functional properties [44], there is still a lack of knowledge regarding the LMSC subpopulations and their characteristics. Furthermore, cellular therapy often requires cells to be expanded to reach a sufficient number before transplantation treatments, [59,60] and the gene expression profiles of MSCs can change during subculture [61].

Therefore, this study aimed to investigate the heterogenicity of LMSCs isolated from the limbal niche and identify possible sub-populations based on fifteen membrane molecules previously described to be present in MSCs by Peng et al. [44], including typical MSC markers, wound healing-associated markers, immune regulation markers, ASC and endothelial-specific markers, and differentiation-associated markers. Furthermore, VEGF, VEGF-R2, and PEDF gene expression rates were assessed to evaluate the angiogenic and antiangiogenic profiles of LMSCs.

## 2. Results

### 2.1. Cell Proliferation and Morphology

**Figure 1:**
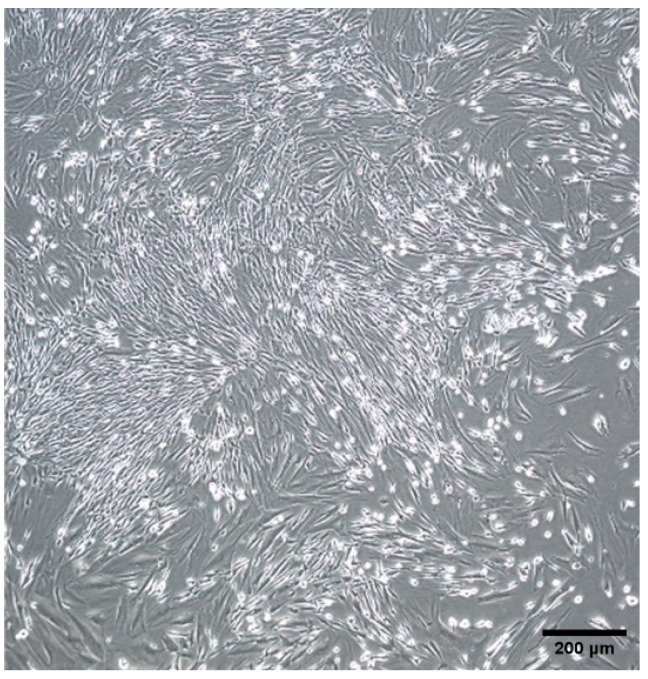
Limbal mesenchymal stromal cell (LMSC) morphology in passage 1. Original magnification 4×.

Following isolation, the LMSCs that seeded and expanded at standard cell culture conditions for eight passages demonstrated a mean ± SD cell doubling time of 1.84 ± 0.08. The cells formed a monolayer over an average time of two weeks and presented a morphology typical of fibroblasts, including an elongated or spindle shape with a single nucleus (Figure 1).

### 2.2. Surface Markers Expression

**Figure 2:**
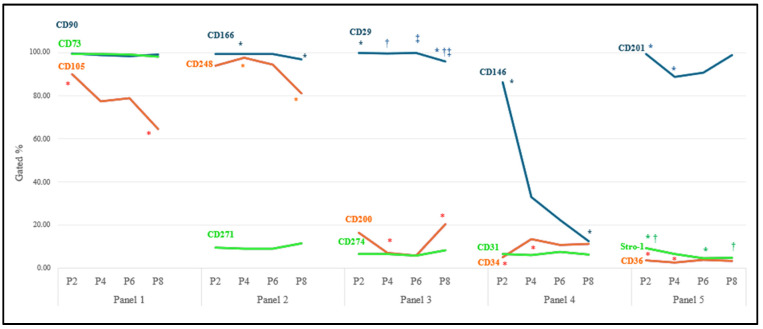
Immunophenotypic analysis of LMSCs. Different surface cell markers were expressed in LMSCs over eight passages (*n* = 6). Mean expression of single cell markers over eight passages in limbal mesenchymal stromal cells. Pairwise significant differences (*p* ˂ 0.05) in each row are demonstrated with *, †, and ‡. Standard deviations can be found in Table S1.

No statistically significant difference was seen between the 15 CD markers’ gene expression at the early stage and during cell expansion among the different donors. The results demonstrated various expressions among the different surface markers over the subculture (Figure 2, Table S1). CD90 and CD73 from Panel 1, CD166 from Panel 2, and CD 29 from Panel 3 indicated a high, consistent, and uniform expression, while CD36 and Stro-1 were expressed in a very low (<10%) but stable population of the LMSCs throughout the culture period. On the other hand, the expressions CD105, CD201, and CD248 demonstrated a gradual decline with sub-cultivation while staying above 50 percent of the heterogenous cell population. The CD146 in Panel 4 showed a decreasing trend from P2 to P8, with this difference appearing significant between P2 and P8. Other markers, including CD34 and CD31 from Panel 4, CD200 and CD274 from Panel 3, and CD271 from Panel 2, also demonstrated subpopulations with different expression profiles, with none being above 20 percent (Figure 2).

**Figure 3:**
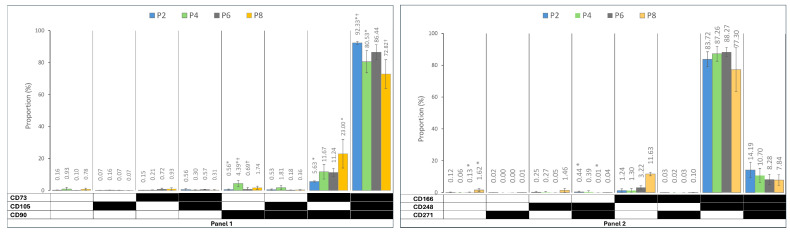

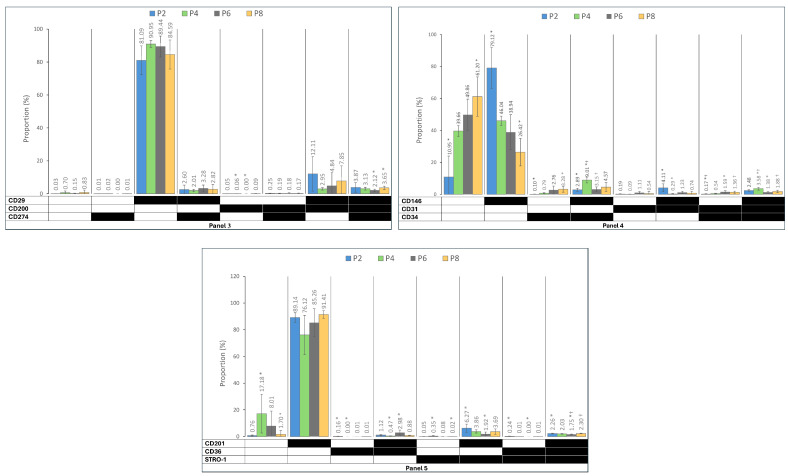
The prevalence of immunophenotypes within the five functional panels from passages 2 to 8 (P2–P8). Panel 1: MSC markers, Panel 2: wound healing markers, Panel 3: immune regulation markers, Panel 4: ASC and endothelial cells’ markers, and Panel 5: differentiation capacity markers. The black field shows that the marker was expressed, and the white field indicates that the marker was not expressed. The data are presented as mean± standard deviations. (* †): indicates a statistically significant change *p*-value < 0.05, which was adjusted by the Bonferroni correction for multiple tests.

Figure 3 displays the prevalence of immunophenotypes and different co-expression profiles in each panel during the subculture. While the spectrum of heterogenous sub-populations identified based on triple marker combinations was not very large, early passages demonstrated significantly dominant populations of CD90+CD73+CD105+ in Panel 1, with the CD73+CD105−CD90+ population showing a significant increase in culture from P2 to P8. In Panel 2, while CD166+CD248+CD271− cells were dominant over the subculture period, CD166+CD248−CD271− demonstrated a significant increase from P2 to P8. Panel 3 showed a constantly dominant population of C29+CD200−CD274− throughout the culture. In Panel 4, CD146+CD31−CD34− was the dominant population at P2, replaced with CD146−CD31−CD34− cells at P8. Panel 5 revealed the CD201+CD36−Stro1− to be dominant throughout the subculture period, with the CD201−CD36−Stro1− population showing a significant increase from P2 to P4, followed by a decreasing trend to P8.

### 2.3. Pro- and Anti-Angiogenic Gene Expression

**Figure 4:**
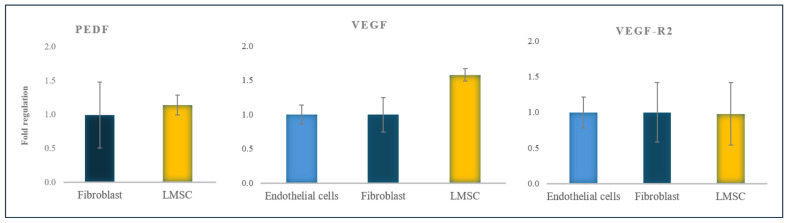
PEDF, VEGF, and VEGF-R2 gene expression ratio in LMSCs normalized to fibroblasts and endothelial cells (all from the 6th passage). The data are presented as mean + standard deviations (SDs).

The results were assessed using the Pfaffl method and indicated higher expression of VEGF in LMSCs compared to fibroblasts and endothelial cells, although the expression ratio of VEGF-R2 in LMSCs was relatively similar to fibroblasts and endothelial cells. PEDF expression in LMSCs was relatively similar to fibroblasts, and based on its anti-angiogenic role, the expression of PEDF was not observed in endothelial cells (Figure 4).

## 3. Discussion

MSCs demonstrate promising potential for the regeneration of ophthalmologic disorders in clinical trials, including dry eye disease [38] and keratoconus [62]. Although ASCs are the primary MSC source used in current studies [40], LMSCs may be a better candidate. Originating from the limbal niche, LMSCs could possess unique properties that make them more suitable for ophthalmologic regeneration.

Various studies have characterized LMSCs using CD90, CD73, and CD105 as notable positive markers [53,55,63,64]. Additionally, CD29 and CD166 have been identified as positive markers for LMSCs [54,65]. Conversely, some investigations did not differentiate the limbus from the cornea, which is not referenced in the current study due to differences in the expression of markers such as CD34 and CD146 [66].

In the current study, the CD markers were expanded to 15 different markers and divided into different panels based on their functionalities, which provides a more comprehensive overview of their roles and credible functions. The mean cell doubling time of the LMSCs over eight passages (1.84 ± 0.08) indicated a robust proliferative capacity comparable to other MSC types (e.g., adipose tissue-, Wharton’s jelly, and bone marrow-derived types) reported in previous studies [67]. The sustained proliferative rate over these eight passages suggests that LMSCs can maintain their viability and replicative potential during extended culture periods, which is crucial for their utility in research and therapeutic applications.

The expression of surface cell markers in MSCs can vary depending on their source [68,69]. The assessment of MSCs using a broader range of markers by Peng et al. revealed a mixture of diverse phenotypes that changed during subculture [44,70]. In this study, Panel 1 (typical MSC surface markers) aimed to validate the identity of the isolated cells as MSCs by confirming the concurrent expression of CD90, CD73, and CD105 as the conventional surface cell markers for MSCs [53]. The findings reveal a high expression of CD73, CD90, and CD105 (Panel 1) and the absence of hematopoietic stem cell markers (CD45 and CD31) as the recognized hallmark of MSC characteristics, validating the identity of the isolated cells.

However, the necessity for the simultaneous expression of all three markers (CD90, CD73, and CD105) precludes their utility in categorizing these cells into distinct subpopulations. Therefore, Panels 2–5 aimed to categorize the cells into four distinct subpopulations based on markers representing different functional capabilities, including wound healing (Panel 2), immune regulation (Panel 3), ASC and endothelial markers (Panel 4), and differentiation capacity (Panel 5) over different passages.

As for the other markers evaluated in this study, CD271 is conventionally utilized as a suitable marker for MSC identification and isolation [68]; its expression is significantly different among various sources with BM-MSCs [71] and ASCs [72] have a higher expression than other MSC sources [73]. Our results also show a low expression of CD271, reducing its efficacy as an identification marker for LMSCs, especially in the early stages of culture. Furthermore, the expression of CD200 [74] and CD274 [75] in MSCs positively correlates with their immunomodulatory abilities, although they predominantly express these markers. Therefore, the differences between our findings and the previous studies could be attributed to the varying expression of MSC surface markers based on their sources.

The notable expression of CD166 and CD248 wound healing markers [76,77,78] throughout the eight passages highlighted the role of LMSCs in the ocular surface wound healing process, while the continuous presence of the CD166+CD248+CD271− subpopulation over the passages established the stability of this subpopulation.

The role of LMSC in acute ocular surface wound healing, as suggested by previous studies [79,80], could contribute to this sub-population. It is also reported that following injury, LMSCs release various growth factors and facilitate interactions among adhesion molecules, growth factors, ECM proteins, and cytokines, resulting in LMSCs producing and secreting a suitable number of paracrine factors, like TGF-β and FGF, that provide wound healing [53,81].

Panel 3 revealed the majority of the cells (>80%) to be CD29+CD200−CD274− throughout the culture. Despite the lower (<20) and negative (<10) expression of CD200 and CD274, the robust expression of CD29 indicated that LMSCs could play an immunomodulatory role, confirming previous studies on ASCs [44] and BM-MSCs [82]. However, the prolongation and extent of this protective role in vivo and under chronic conditions remain unclear.

In Panel 4, the CD146+CD34−CD31− cell population comprised the majority of cells, indicating a predominance of MSC-associated characteristics [67] and the absence of endothelial and hematopoietic stem cells [83,84,85] during the early stages (P2 and P4); as a study by Liu et al. indicated, LMSCs exhibit high expression of CD146 at a similar stage [66]. However, the expression of CD146 gradually decreased over culture, resulting in a significantly higher and more homogeneous sub-population of CD146−CD34−CD31− cells at P8, which are less associated with endothelial and vascular smooth muscle cells [84,85,86]. CD34 expression was negative in P2 and, despite the significant increase in P4, remained consistently low until the eighth passage, not affecting the cell populations categorized based on the co-expression of markers in the panel.

Differentiation potential into other cell lineages is one of the most important characteristics of MSCs, which can exhibit varying expression patterns and differentiation potentials depending on their source [87]. The results from Panel 5 indicate a uniform expression of the CD201+CD36−Stro-1 subpopulation throughout the sub-culture, which may be attributed to the source of MSCs and their potency.

Higher CD201, CD36, and Stro-1 expression levels have been reported in umbilical cord-derived MSCs [87] but not in other MSC sources. Stro-1 levels have been reported to be notably low compared to other positive markers, with a previous study showing only about 9% of passaged MSCs expressing Stro-1 [88], which corresponds to P2 in this study. On the other hand, the significant drop in Stro-1 from P2 to P8 represents decreased differentiation potential with aging, as reported by other studies [61,89]. Providing the differentiation circumstances is necessary to investigate the more specific differentiation potentials of LMSCs with further aging. Future studies are warranted to provide a comparative assessment of the marker expressions between MSCs from different sources and other cell types.

Fibroblasts, as mesenchymal-derived cells, significantly contribute to angiogenesis through the mesenchymal-to-endothelial transition [90]. The notable expression of endothelial cell markers, including CD29, CD 201, and CD248 [91], observed in the LMSCs suggests that they may also have the potential to differentiate into endothelial cells, which are capable of inducing ocular surface angiogenesis and play a crucial role in corneal neovascularization.

VEGF (vascular endothelial growth factor) is a potent proangiogenic factor that binds the VEGF-R2 receptor on endothelial cells, activating intracellular signaling pathways that stimulate cell proliferation, migration, survival, and promote angiogenesis [92], which can also result in corneal vascularization [93].

PEDF is a glycoprotein growth factor released by limbal stromal cells that maintains the survival and multipotency of LESCs and promotes wound healing [94,95]. PEDF is considered the key regulator of the corneal angiogenic privilege, playing its anti-angiogenic role through directly binding to VEGF-R2, inhibiting VEGF from engaging and activating the receptor, thus inhibiting VEGF-induced angiogenesis [96]. Additionally, PEDF can induce apoptosis in proliferating endothelial cells, further suppressing angiogenesis [97].

A study on ischemia-induced retinal neovascularization demonstrated that VEGF levels were increased to a greater extent than PEDF (as stimulators and inhibitors of angiogenesis, respectively). This VEGF–PEDF ratio-dependent retinal neovascularization suggests that an impaired VEGF–PEDF balance is the key contributor to retinal neovascularization [98]. More recent studies have also confirmed that balancing the proangiogenic effects of VEGF and the anti-angiogenic effects of PEDF is crucial in regulating angiogenesis [92,97].

The corneal angiogenic privilege can be severely impaired by inflammatory and infectious disease of the ocular surface [99]. This could be due to the release of proangiogenic cytokines, including VEGF, by several cell types, including corneal epithelial cells, corneal and vascular endothelial cells, immune cells (e.g., T-cells and macrophages), pericytes and stromal keratocytes following inflammatory corneal tissue injury [93].

This study evaluated the expression of VEGF, VEGF-R2, and PEDF in LMSCs compared to endothelial cells and fibroblasts (as positive and negative controls, respectively) to better understand their angiogenic potential. The similar expression of VEGF-R2 in endothelial cells, LMSCs, and fibroblasts found in this study suggested that the limbal cells could differentiate into endothelial cells, bind to VEGF, and initiate angiogenesis via the VEGF/VEGF-R2 signaling pathway. The higher expression of VEGF in LMSCs, compared to endothelial cells and fibroblasts, further supports their involvement in neovascularizing the ocular surface.

We observed a high expression of PEDF in fibroblasts and LMSCs but a lower expression in endothelial cells, consistent with previous studies [99,100,101,102]. The prominent expression of PEDF in fibroblasts has been linked to their anti-inflammatory [95,102,103] and anti-angiogenic [104] properties. Our findings revealed a similar PEDF expression level in LMSCs and fibroblasts, suggesting that the LMSCs may also play an anti-inflammatory and anti-angiogenic role in the healthy limbus, potentially mitigating the effects of pro-inflammatory cytokines generated by external stimuli.

## 4. Materials and Methods

Remnants of anonymized corneal transplant specimens used for posterior lamellar keratoplasty from donors (aged 30–70) without any corneal disease were obtained from the Danish Cornea Bank (Aarhus University Hospital, Aarhus, Denmark) via the applicable Danish legislation. The specimens were stored in a specific organ-culture storage media to preserve their viability.

Following the isolation and establishment of LMSC primary cultures, the cells were sub-cultured for eight passages (P). Flow cytometric analysis was used to assess the expression and co-expression of the surface markers distributed in five panels in cells from P2, P4, P6, and P8. Panel 1 included CD90, CD105, and CD73 (typical MSC markers); panel 2 comprised CD166, CD248, and CD271 (wound healing associated sub-population); panel 3 included CD29, CD274, and CD200 (immune regulation associated sub-population); Panel 4 contained CD146, CD34 and CD31 (ASC and endothelial-specific markers); and panel 5 included CD201, CD36, and Stro-1 (differentiation associated sub-population) (Table 1).

qPCR analyses were used to evaluate the expression of the pigment epithelial-derived factor (PEDF), vascular endothelial growth factor (VEGF), and vascular endothelial growth factor receptor (VEGF-R2) genes in LMSCs (P6), and dermal fibroblasts (P6) and vascular endothelial cells (P6) as comparators.

### 4.1. Cell Culture

To establish the primary culture of LMSCs, the cornea was removed by trephine, and the limbus was isolated from the remaining tissue, cut into 1–2 mm pieces using a scalpel blade, and enzymatically digested by suspending in 1 mL of 2 mg/mL collagenase (Roche Diagnostics, Indianapolis, IN, USA) for one hour at 37 °C. The resulting cell clusters, comprising LESC-LMSC-LM, were collected using reversible cell strainers with a pore size of 37µm (BD Falcon, Franklin Lakes, NJ, USA) and were further dissociated into single cells by digesting in 1 mL of 0.25% trypsin and 0.02% EDTA (Gibco, Taastrup, Denmark) at 37 °C for 15 min. Single-cell suspensions were seeded into T25 flasks (Greiner Bio-one, Frickenhausen, Germany) and cultured in MEM alpha (Gibco, Taastrup, Denmark) supplemented with 10% FCS (Gibco, Taastrup, Denmark) and 1% Penicillin/Streptomycin (Gibco, Taastrup, Denmark). The medium was changed every other day until the cells reached 80% confluency. At this point, the cells were sub-cultured. They were rinsed twice with 1× sterile PBS (phosphate-buffered saline) (Gibco, Taastrup, Denmark) to remove dead cells and debris. Subsequently, the cells were treated with an appropriate amount of TrypLE (Gibco, Taastrup, Denmark) based on the flask size to detach the cells. The enzyme activity was neutralized by adding twice the volume of TrypLE to the media. The cell suspension was then centrifuged at 500× *g* for 5 min. After removing the supernatant, the cells were transferred to a T75 flask (Greiner Bio-one, Frickenhausen, Germany). The cells were sub-cultured for up to 8 passages.

MEM alpha only supports the growth of MSCs and fibroblasts, while other limbal niche cells require supplementary growth factors. Nevertheless, flow cytometry assessments were performed to ensure the purity of the cell population in passage I. However, dermal fibroblast cells (GM08680) were cultured in MEM alpha (Gibco, Taastrup, Denmark) supplemented by 10% FCS (Gibco, Taastrup, Denmark) and 1% Penicillin/Streptomycin (Gibco, Taastrup, Denmark). Endothelial cells were cultured in the growth medium MV2 (Promo Cell, Germany) with its specific supplement. Dermal fibroblasts and endothelial cells were both sub-cultured to P6 for RT-qPCR analysis.

### 4.2. Multichromatic Flow Cytometry

The 15 directly conjugated antibodies (BD Bioscience, Lyngby, Denmark) together with fixable viability stain 570 (FVS 570) (BD Bioscience, Lyngby, Denmark) were assigned into five panels, as shown in Table 2. For the staining procedure, cells were initially incubated with the viability dye for 15 min at room temperature. Following this, a mixture of antibodies, optimally diluted in PBS supplemented with 2% FCS and 0.1% sodium azide (Merck Schuchardt, Hohenbrunn, Germany), was applied for 30 min at 4 °C in the dark.

All buffers used in staining were sterile PBS-based supplies with 50% Accumax (Sigma-Aldrich, St. Louis, MI, USA) and 25 nM HEPES (Life Technologies, Carlsbad, CA, USA) to maintain the appropriate PH range and prevent cell clumping. Firstly, the cells were incubated with the viability reagent for 15 min at room temperature before the suspensions were stained for surface antibodies, which were optimally diluted in PBS supplemented with 2% FCS and 0.1% sodium azide (Merck Schuchardt, Hohenbrunn, Germany) and incubated for 30 min at 4 °C in the dark. Finally, the stained cells were transferred into a 5 mL round-bottom glass FACS tube (BD Falcon, Albertslund, Denmark) for surface epitope analysis using the CytoFLEX (Beckman Colter, Copenhagen, Denmark) flow cytometer.

Before analysis, compensation values were established with the aid of the BD CompBeads Plus Set Anti-Mouse Ig, κ, and Anti-Rat Ig, κ (BD Biosciences, NJ, USA). The data were analyzed using the Kaluza 2.1 software (Beckman Coulter, Indianapolis, IN, USA). Basic gates were applied to target alive singlets, and the top 2.5 percentile of unstained cells (fluorescence minus one (FMO) control) was regarded as positive. Tree plots were used to visualize the subpopulations. Figure S1 illustrates a representative example of the gating strategy used for the flow cytometric analysis.

### 4.3. Real Time-qPCR

RNA isolation from LMSC was performed using the Aurum Total RNA Mini Kit (Bio-Rad, USA), while the purity and concentration of RNA were determined using a nanoliter spectrophotometer (NanoDrop; Thermo Fisher Scientific, Waltham, MA, USA) and first-strand cDNA synthesis was performed using RNA from lysed cultured cells and the iScript™ reverse transcriptase kit (Bio-RAD, Hercules, CA, USA). PCR reactions were performed in a CFX Connect Real-Time PCR instrument (Bio-Rad, USA), as they contained target-specific primers (Table 3), the IQ SYBR Green Supermix (Bio-Rad), and cDNA according to the manufacturer’s instructions. PPIA (Peptidylprolyl isomerase A) was utilized as the housekeeping gene. Gene expression levels and ratios were normalized relative to PPIA and comparatively evaluated using ΔΔC_T_ or Pfaffl methods.

### 4.4. Statistical Analysis

The Shapiro–Wilks test was used to check for normal distribution. Unless otherwise stated, data are presented as the mean ± standard deviation (SD). Friedman’s related-samples two-way Analysis of Variance by ranks was utilized to investigate the differences between different groups at different passages. *p* < 0.05 was considered significant, while the Nonparametric Test procedure with repeated measures was applied, and significance values were adjusted by the Bonferroni correction for multiple tests.

## 5. Conclusions

In conclusion, the LMSCs proliferated robustly over eight passages, indicating their viability for extended culture and potential for therapeutic use. Surface marker analysis confirmed the identity of LMSCs as MSCs with distinct subpopulations. The significant expression of CD166 and CD248 markers associated with wound healing suggested that LMSCs may play a role in ocular surface repair. The tendency towards higher VEGF expression in LMSCs than in fibroblasts and endothelial cells indicated that LMSCs could also contribute to ocular surface regeneration through paracrine actions, modulating angiogenesis and inflammation. Additionally, CD29 expression profiles revealed an immunomodulatory role. The comparable PEDF expression in LMSCs and fibroblasts further supported the anti-inflammatory and anti-angiogenic role of LMSCs. The stability of the CD201+CD36-Stro1- subpopulation over passages indicated the differentiation capability of LMSCs, while the expression of VEGF-R2 could indicate the LMSC-to-endothelial cell differentiation ability.

This study comprehensively characterized LMSC subpopulations based on surface cell markers, emphasizing their heterogeneity and functional potential in regenerative therapies for ocular surface disorders. Identifying distinct LMSC subpopulations based on surface markers and gene expression profiles lays the groundwork for standardized clinical protocols. Future research should focus on elucidating the in vivo behavior of these subpopulations and their long-term effects under pathological conditions.

## Figures and Tables

**Table 1:** Panel design for flow cytometry. Cell surface markers and their functions in each panel.

Panels	CD Markers	References
Panel 1	CD 90	[44,105,106,107]
	CD105	[107,108,109]
	CD73	[110,111]
Panel 2	CD166	[106,112]
	CD248	[106,113,114,115]
	CD271	[73]
Panel 3	CD29	[106,116]
	CD200	[117,118]
	CD274	[119,120]
Panel 4	CD146	[106,121]
	CD34	[122]
	CD31	[107,123]
Panel 5	CD201	[106,124]
	CD36	[125,126]
	STRO-1	[106,127]

**Table 2:** Cytometer set up for 15 markers. BP: band pass, FVS570: fixable viability stain 570, and AF647: Alexa Fluor 647.

Antibody	Emission Channel	Fluorochrome	Laser
CD201	450/45 BP	BV421	405 nm
CD105	525/40 BP	BV510	
CD 166, CD36	610/20 BP	BV605	
CD29	660/20 BP	BV650	
CD73	525/40 BP	FITC	488 nm
CD90	690/50 BP	PerCP-Cy5.5	
CD146	610/20 BP	PE-CF594	561 nm
FVS570	585/42 BP	Viability dye	
CD271, CD200, CD34	780/60 BP	PE-Cy7	
CD248, STRO-1	660/20 BP	AF647	638 nm
CD274	712/25 BP	APC-R700	
CD31	780/50 BP	APC-Cy7	

Panel 1: MSC markers, Panel 2: Wound healing markers, Panel 3: Immune regulation markers, Panel 4: ASC and endothelial cell markers, and Panel 4: Differentiation potency markers. ASCs: Adipose-derived stem cells; MSCs: mesenchymal stem/stromal cells.

**Table 3:** Primers sequences applied in RT-qPCR.

Gene Symbol	Primer Sequences	
*PPIA*	Forward	5′ TCC TGG CAT CTT GTC CAT G 3′
	Reverse	5′ CCA TCC AAC CAC TCA GTC TTG 3′
*VEGF*	Forward	5′ CAT TGA TCC GGG TTT TAT CC 3′
	Reverse	5′ CGA TTC AAG TGG GGA ATG G 3′
*VEGF-R2*	Forward	5′ CAG CAG GAT GGC AAA GAC TAC A 3′
	Reverse	5′ GGC AGA GAG AGT CCA GAA TCC TC 3′
*PEDF*	Forward	5′ TGT GCA GGC TTA GAG GGA CT-3′
	Reverse	5′ GTT CAC GGG GAC TTT GAA GA-3′

## Data Availability

The data supporting this study’s findings are available from the author, [S.A.], upon reasonable request.

## Supplementary Materials

The supporting information can be downloaded at https://www.mdpi.com/article/10.3390/ijms25168684/s1.
