# Supplementary material for: Immunophenotypical Characterization of Limbal Mesenchymal Stromal Cell Subsets during In Vitro Expansion
Source: Int J Mol Sci. 2024 Aug 9;25(16):8684. doi: 10.3390/ijms25168684 (PMC11354999; doi:10.3390/ijms25168684)
Supplement: Supplementary file 1 [file ijms-25-08684-s001.zip › ijms-3115152-supplementary.pdf]

Table S1. Mean ± SD expression of single cell markers over eight passages in limbal mesenchymal stromal cells. Pairwise significant differences ($p<0.05$) in each row have been demonstrated with *, †, and ‡. MSC: mesenchymal stem cell; WH: wound healing; IR: immune

Panels	Surface Markers	Passage 2	Passage 4	Passage 6	Passage 8
**Panel 1**	CD 90	99.48 ± 0.67	98.9 ± 1.32	98.33 ± 1.52	99.13 ± 0.70
**(MSC markers)**	CD105	89.91 ± 4.70 *	77.33 ± 8.67	78.83 ± 7.75	64.53 ± 10.94*
	CD73	99.38 ± 0.70	99.4 ± 0.32	98.96 ± 1.09	98.05 ± 1.45
**Panel 2**	CD166	99.20 ± 0.66	99.32 ± 1.04 *	99.33 ± 0.85	96.85 ± 1.66 *
**(WH markers)**	CD248	93.77 ± 4.04	97.63 ± 3.45 *	94.37 ± 3.87	81.08 ± 9.33 *
	CD271	9.43 ± 3.20	9.15 ± 1.73	8.97 ± 1.34	11.53 ± 2.55
**Panel 3**	CD29	99.75 ± 0.16 *	99.55 ± 0.46 †	99.73 ± 0.26 ‡	95.88 ± 4.63 *, †, ‡
**(IR markers)**	CD200	16.43 ± 7.26	6.98 ± 2.19	5.87 ± 4.15 *	20.28 ± 5.11 *
	CD274	6.51 ± 2.75	6.60 ± 2.61	5.80 ± 2.29	8.27 ± 1.63
**Panel 4**	CD146	86.18 ± 13.97*	32.93 ± 12.87	22.45 ± 6.09	12.47 ± 5.10 *
**(EC, ASC**	CD34	5.09 ± 1.03 *	13.43 ± 1.02 *	10.77 ± 3.00	11.23 ± 2.37
**markers)**	CD31	6.48 ± 1.91	6.22 ± 1.89	7.50 ± 2.50	6.28 ± 2.61
**Panel 5**	CD201	99.20 ± 0.24 *	88.63 ± 6.39 *	90.55 ± 13.05	98.80 ± 0.43
**(DC markers)**	CD36	3.75 ± 0.37 *	2.55 ± 0.20*	3.80 ± 1.68	3.50 ± 0.37
	STRO-1	9.33 ± 0.65 *, †	6.55 ± 1.16	4.53 ± 1.09 *	4.83 ± 1.13 †

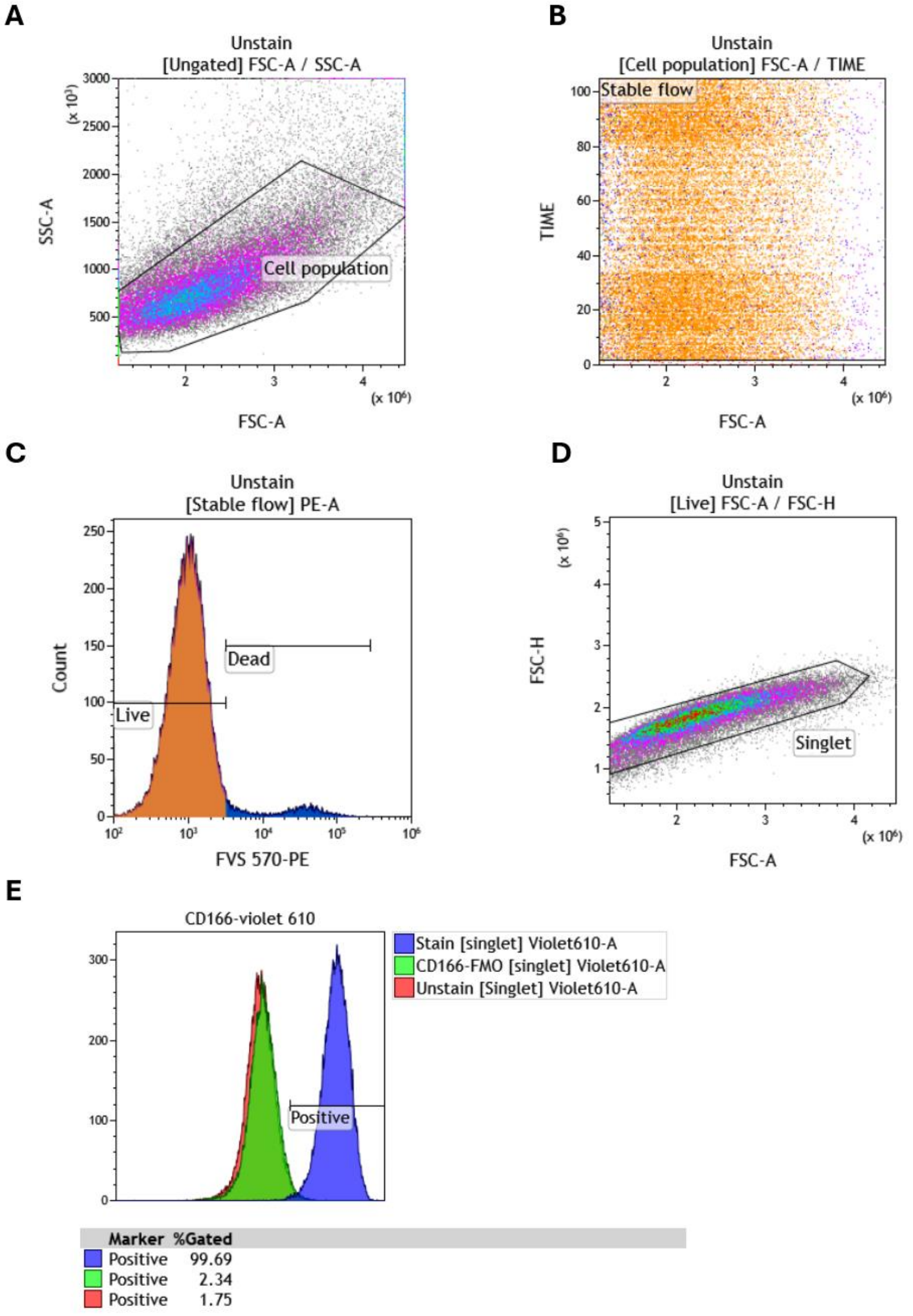

Figure S1. Gating strategy for flow cytometry. (**A**) Based on forward scatter area (FSC-A) versus side scatter area (SSC-A) noise was removed to collect the cell population using). (**B**) FSC-A versus time plot assured a stable flow during data acquisition. (**C**) Viability dye (FVS570) was utilized to exclude dead cells. (**D**) The doublets were discriminated via FSC-A versus forward scatter height (FSC-H). (**E**) Representative overlay histogram of the stained, CD166 fluorescence minus one (FMO), and unstained groups. The threshold was set at 2.5% of fluorescence minus one (FMO).
